# Prognosis of olfactory and gustatory dysfunctions in COVID‐19 patients: A case series

**DOI:** 10.1002/ccr3.3269

**Published:** 2020-08-27

**Authors:** Po‐Yu Liu, Rong‐San Jiang

**Affiliations:** ^1^ Division of Infection Department of Internal Medicine Taichung Veterans General Hospital Taichung Taiwan; ^2^ Rong Hsing Research Center for Translational Medicine National Chung Hsing University Taichung Taiwan; ^3^ Department of Medical Research Taichung Veterans General Hospital Taichung Taiwan; ^4^ Department of Otolaryngology Taichung Veterans General Hospital Taichung Taiwan; ^5^ School of Medicine Chung Shan Medical University Taichung Taiwan

**Keywords:** coronavirus disease 2019, gustatory dysfunction, olfactory dysfunction, smell test, taste test

## Abstract

Although most COVID‐19 patients feel their olfactory function returns to normal, the smell test demonstrates that a mild impairment of the olfactory function may have remained. Therefore, their olfactory function should be evaluated by a smell test.

## Graphical abstract

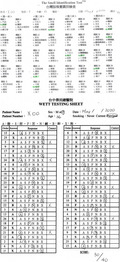

## 1 INTRODUCTION

Olfactory and gustatory dysfunctions are common presentations in COVID‐19 patients. We present three patients who received smell and taste tests after recovery. The smell test suspected persistent impairment of olfactory function in these patients. The report proposes continued evaluation of olfactory function by a smell test in COVID‐19 patients.

The coronavirus disease 2019 (COVID‐19) pandemic currently remains the greatest global health crisis existing today.^1^, ^2^ Olfactory and gustatory dysfunctions have been found to be common presenting symptoms in COVID‐19 patients.^3^ Hyposmia, with or without hypogeusia, has been suggested as a potentially reliable indicator of mild COVID‐19 and is being used in screening for COVID‐19.^4^, ^5^ On the contrary, it has been considered that olfactory and gustatory dysfunctions are self‐limiting in the great majority of COVID‐19 patients.^6^ However, there are rare reports investigating the prognosis of olfactory and gustatory dysfunctions in COVID‐19 patients. Here, we present three patients who suffered from olfactory and gustatory dysfunctions as presenting symptoms of COVID‐19 infection. Their olfactory and gustatory functions were evaluated by the traditional Chinese version of the University of Pennsylvania Smell Identification Test (UPSIT‐TC, Sensonics International) and the Waterless Empirical Taste Test (WETT^®^, Sensonics International) after recovery from COVID‐19.

**Figure 1:**
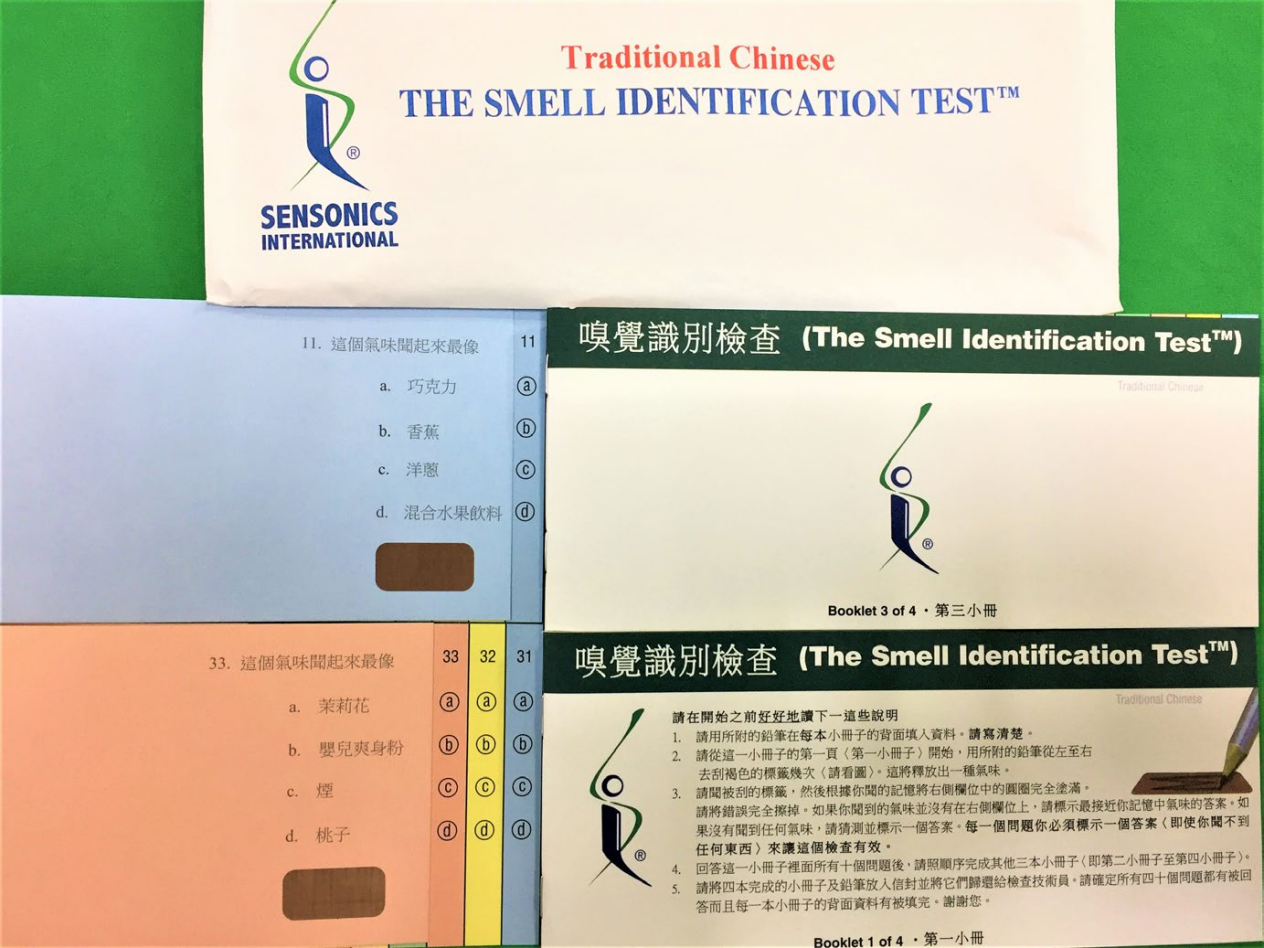
Traditional Chinese version of University of Pennsylvania Smell Identification Test

The UPSIT‐TC is modified from the American version of the UPSIT and is comprised of four 10‐odorant booklets (Figure 1).^7^ Each odorant is embedded in 10‐50 µm microcapsules fixed in a propriety binder and positioned on brown strips at the bottom of the pages of each test booklet.^8^ At the beginning of the UPSIT‐TC, all subjects release each of the 40 odorants by scratching the brown strip with a pencil tip. They are then asked to choose a name from a set of four odor descriptors to identify the released odorant. The test is scored by the number of odors identified correctly to generate a maximum score of 40. An olfactory diagnosis of UPSIT‐TC has been established in relation to gender and age.^9^ For example, the cutoff scores are set at 29.5 between normosmia and mild hyposmia for male adults whose ages range between 20 and 59 years, and are set at 30.5 between normosmia and mild hyposmia for female adults whose ages range between 20 and 59 years.

**Figure 2:**
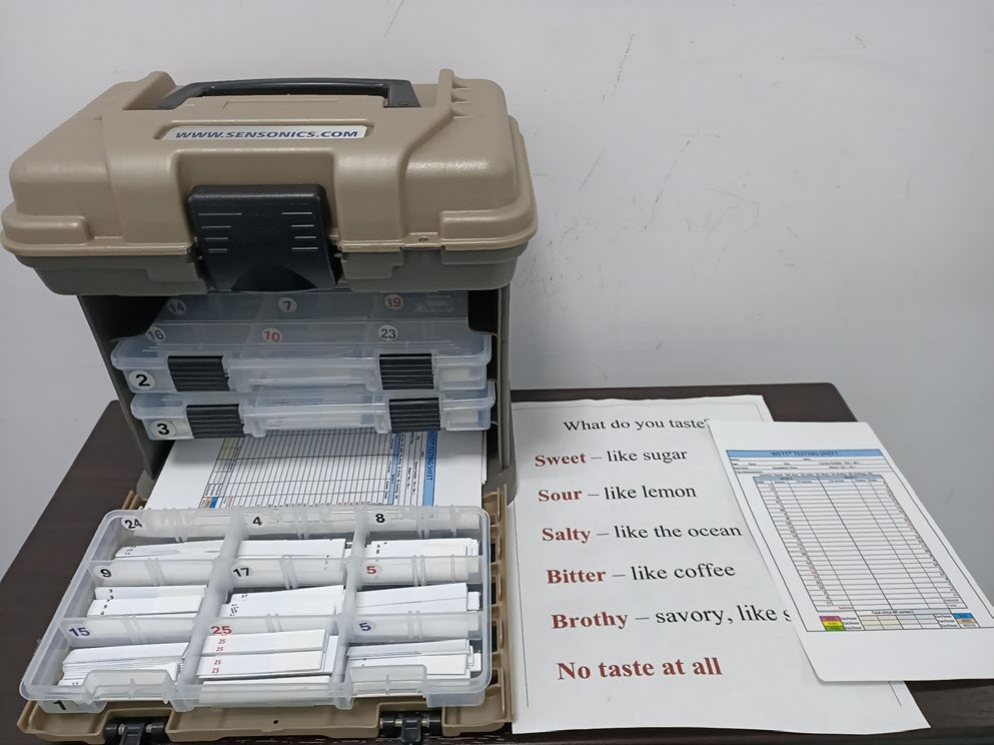
Waterless Empirical Taste Test

The WETT^®^ is comprised of 40 plastic strips with a small pad on the tip of each strip which is embedded with either sucrose, citric acid, sodium chloride, caffeine, or monosodium glutamate tastants. Each tastant contains four different concentrations. The WETT^®^ also incorporates an additional 13 blank strips whose pads are made only of monomer cellulose to make a total of 53 tests (Figure 2). At the beginning of the WETT, all subjects are handed a strip. They place the pad on the strip in the middle of the tongue, close their mouth, and move the strip slightly around.^10^ They are then asked to select one of 6 descriptions (sweet, sour, salty, bitter, brothy, or no taste at all). One point is scored if a correct answer is made, though the scores from the 13 blank strips are not used for analysis of the scoring of the test, thus generating a maximum score of 40 for the test. According to the administration manual, it is possible and not uncommon for subjects with excellent taste to acquire near perfect scores; however, those with normal taste would also attain an average score of around 20.

## 2 PRESENTATION

This case series was conducted on three patients who were diagnosed with COVID‐19 and admitted to Taichung Veterans General Hospital, Taichung, Taiwan. All patients received a UPSIT‐TC and WETT to evaluate their olfactory and gustatory functions after recovery from COVID‐19.

### 2.1 Case 1

A 36‐year‐old healthy woman with a history of allergic rhinitis had suffered from fever episodes while she had been traveling abroad. She did not notice any other symptom except for loss of smell. When she returned home and tested positive for COVID‐19, she was admitted to isolation unit. Her chest X‐ray film revealed lower left lung pneumonia, but the results of her laboratory tests were normal. She was treated with levofloxacin (500 mg QD), hydroxychloroquine (200 mg tid), and azithromycin (500 mg QD). She was discharged from the hospital in stable condition 36 days later after a COVID‐19 RT‐PCR test proved negative three times.

**Figure 3:**
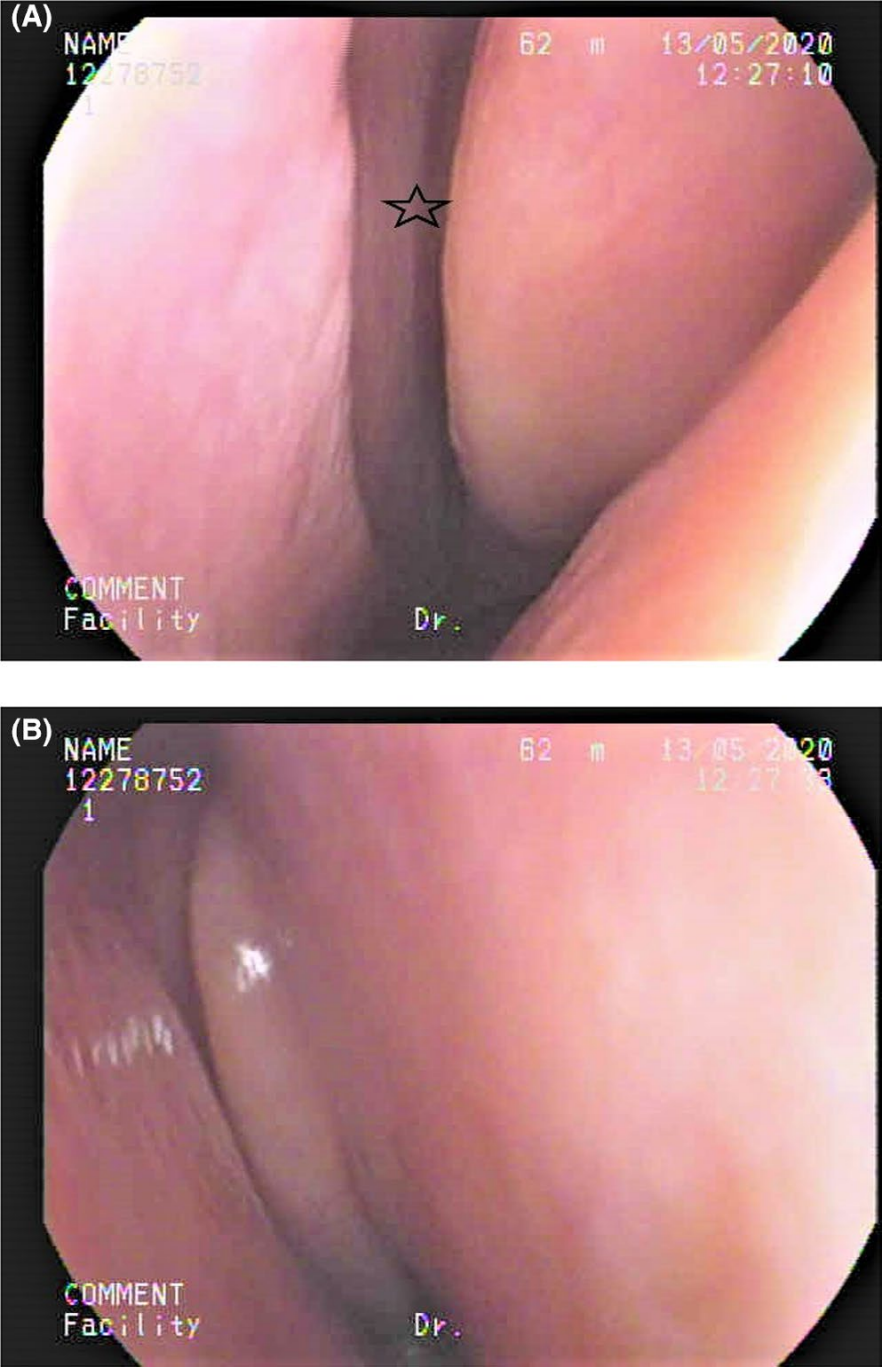
The nasal endoscopy. A, Left olfactory cleft (star) was open. B, Right nasal cavity was clear.

**Figure 4:**
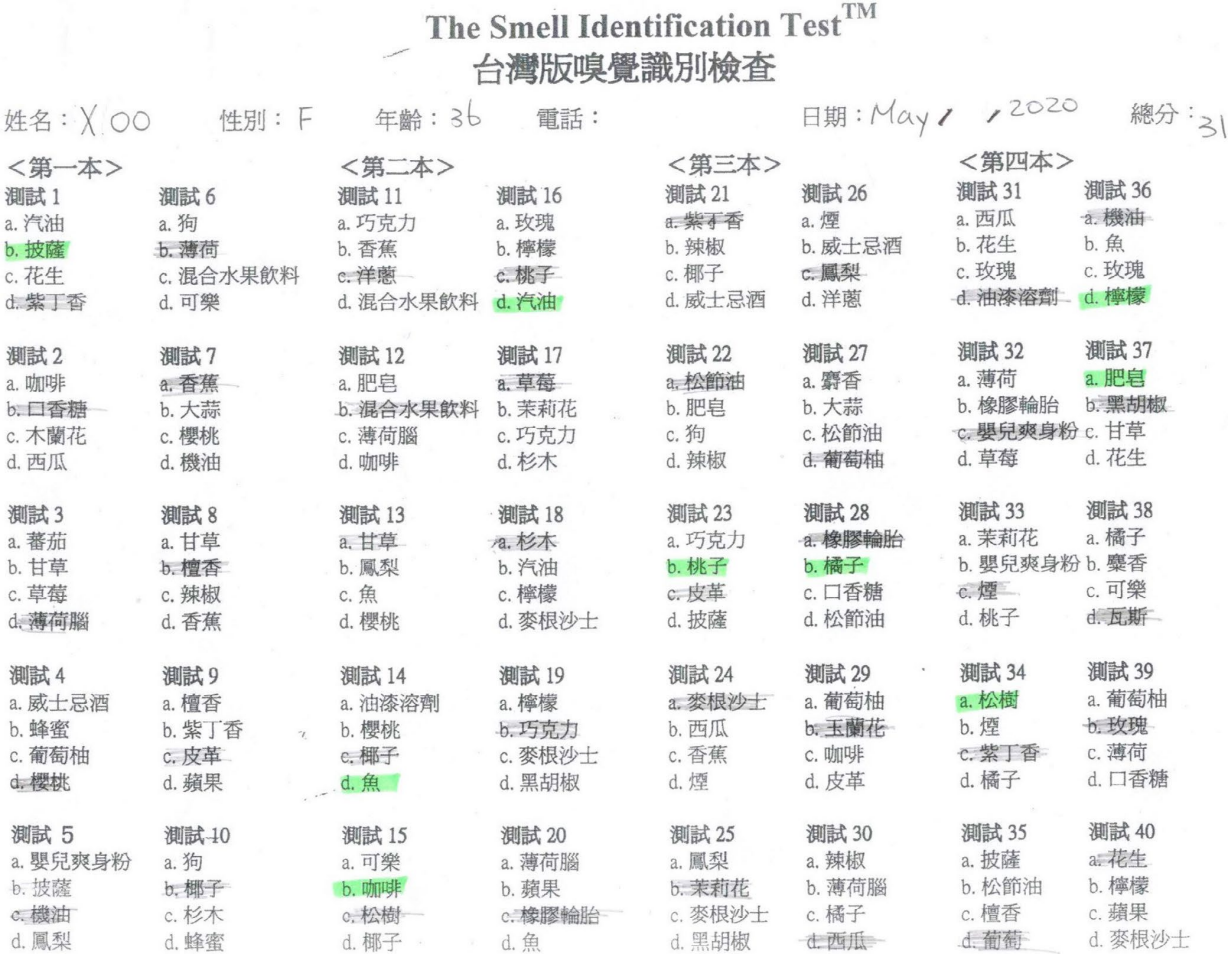
The score of the traditional Chinese version of University of Pennsylvania Smell Identification Test was 31.

**Figure 5:**
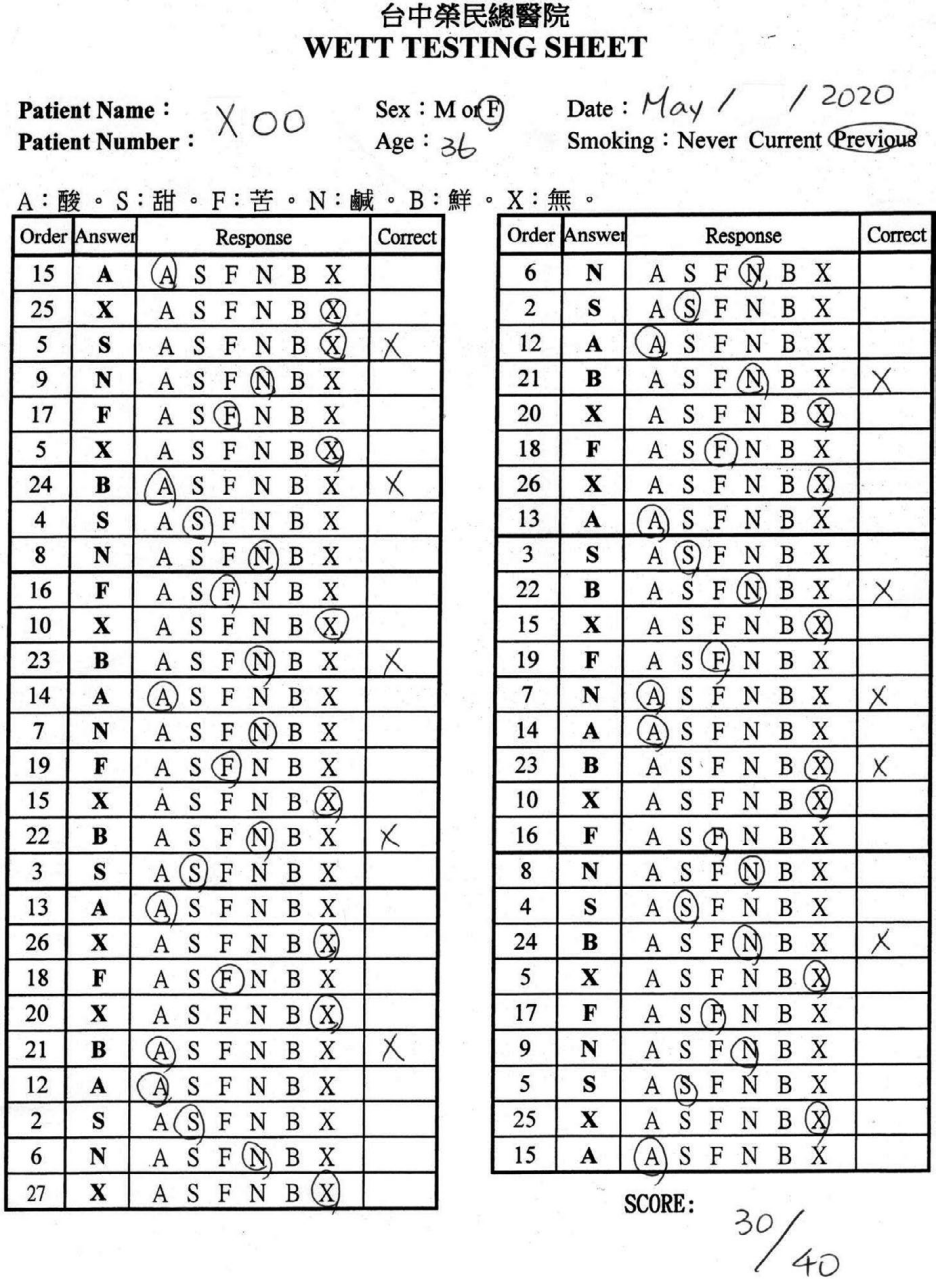
The score of the Waterless Empirical Taste Test was 30.

She followed up with a visit to the Otolaryngology clinic 2 weeks after discharge. She commented that her olfactory function had returned to normal, and her gustatory function was normal. A nasal endoscopy showed the nasal cavity to be free of disease (Figure 3). She received a UPSIT‐TC to evaluate her olfactory function, and the score was 31 (Figure 4). She received a WETT to evaluate her gustatory function and that score was 30 (Figure 5). Without any further treatment, she received another UPSIT‐TC resulting in a score of 28, as well as another WETT resulting in a score of 38 one month later.

### 2.2 Case 2

A 40‐year‐old healthy woman had developed rhinorrhea, hyposmia, and ageusia during self‐isolation at home due to having travelled abroad. She did not have any other symptom. After testing positive for COVID‐19, she was admitted. Her chest X‐ray film showed bilateral increased lung infiltration, while the results of her laboratory tests were normal. She was subsequently treated with both hydroxychloroquine (200 mg tid) and azithromycin (500 mg QD). She noticed that her olfactory and gustatory functions had gradually improved, and was therefore discharged from the hospital in stable condition 20 days later after three COVID‐19 RT‐PCR tests came back negative.

**Figure 6:**
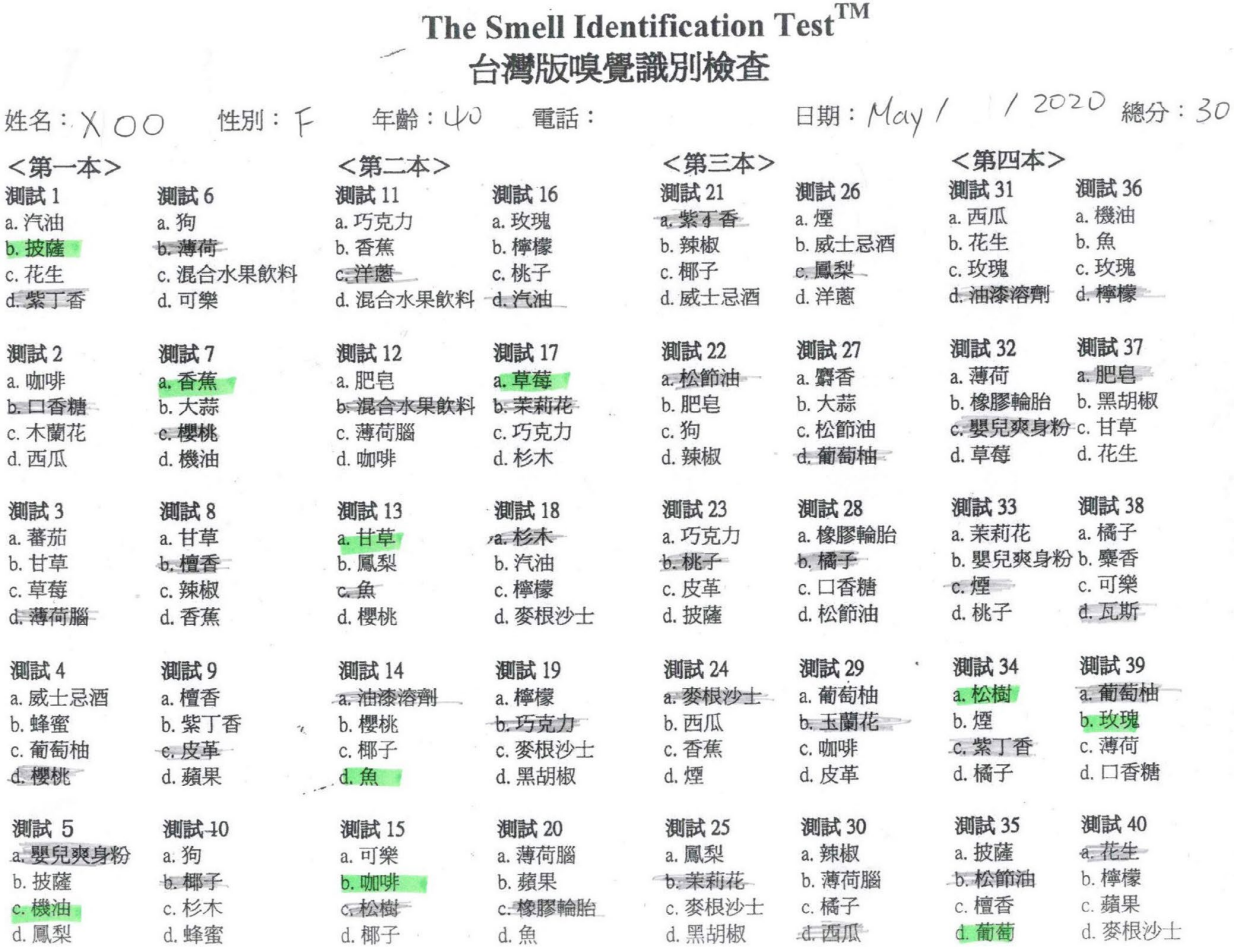
The score of the traditional Chinese version of University of Pennsylvania Smell Identification Test was 30.

**Figure 7:**
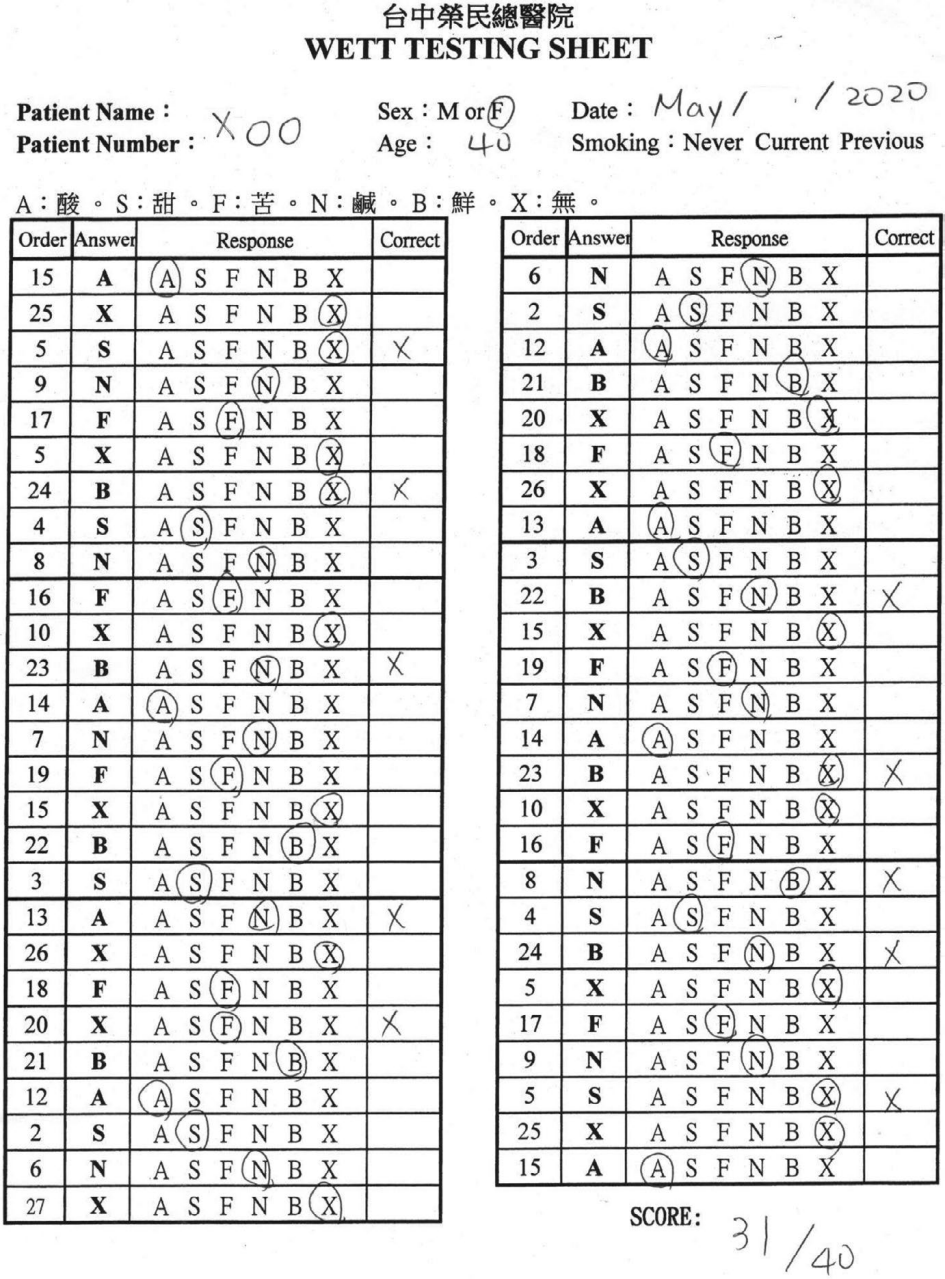
The score of the Waterless Empirical Taste Test was 31.

She later visited the Otolaryngology clinic one month after discharge. She reported that her olfactory and gustatory functions had returned to normal. A nasal endoscopy revealed some watery discharge in the posterior nasal cavity without any sign of other lesions. She received a UPSIT‐TC to evaluate her olfactory function, and her score was 30 (Figure 6). She then received a WETT to evaluate her gustatory function, and the score came back as 31 (Figure 7). Without any further treatment, she received another UPSIT‐TC resulting in a score of 32, as well as another WETT resulting in a score of 38 one month later.

### 2.3 Case 3

A 50‐year‐old diabetic man developed a fever after returning home from traveling abroad. He was also experiencing body aches with fatigue and had noticed a loss of taste. He did not have any other symptom. After testing positive for COVID‐19, he was admitted for isolation. His chest X‐ray film showed bilateral increased lung infiltration, while the results of his laboratory tests were normal, with the exception of a finding of high blood sugar. He was treated with levofloxacin (500 mg QD), hydroxychloroquine (200 mg tid), and azithromycin (500 mg QD). He was discharged from the hospital in stable condition 35 days later after three COVID‐19 RT‐PCR test results came back negative.

**Figure 8:**
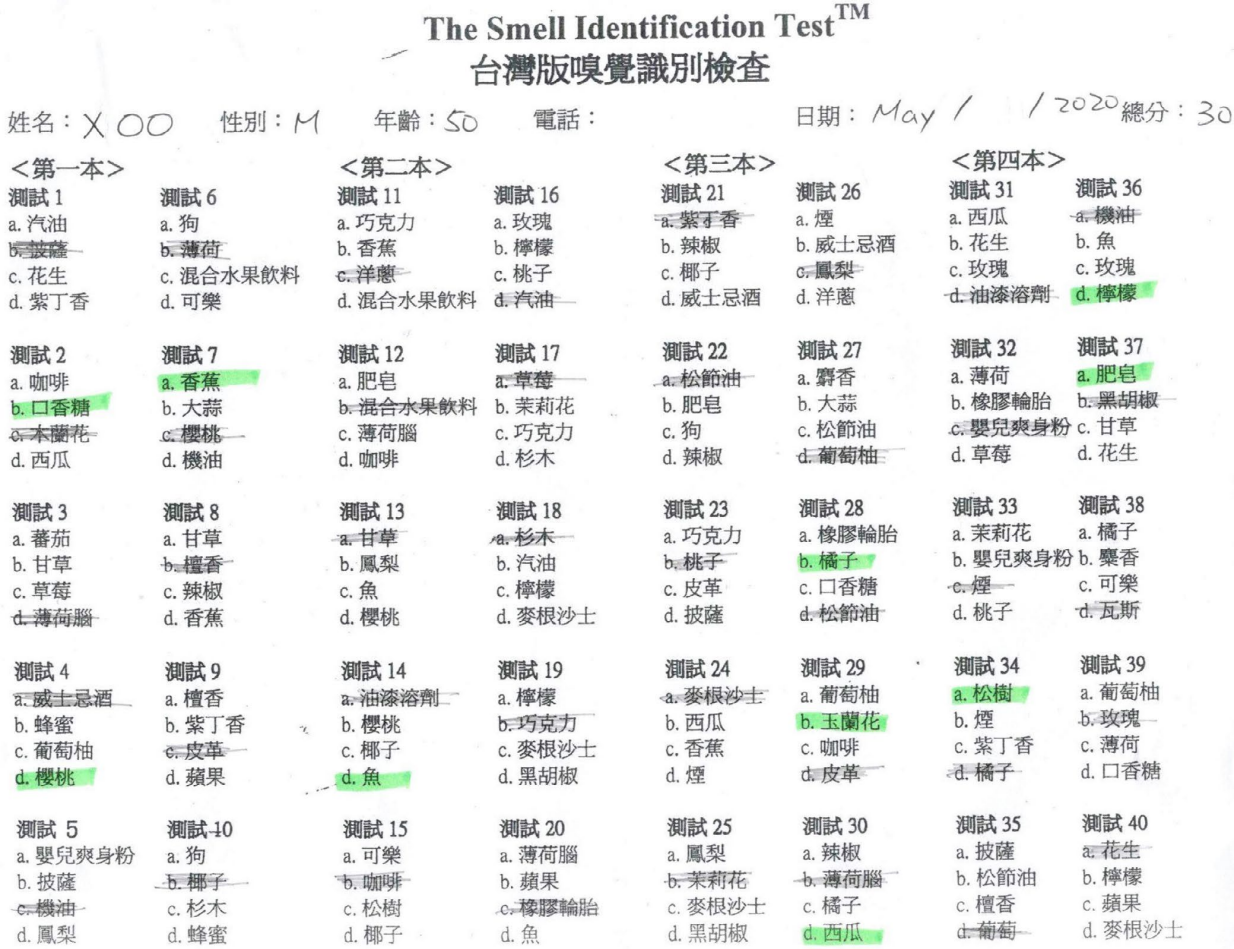
The score of the traditional Chinese version of University of Pennsylvania Smell Identification Test was 30.

**Figure 9:**
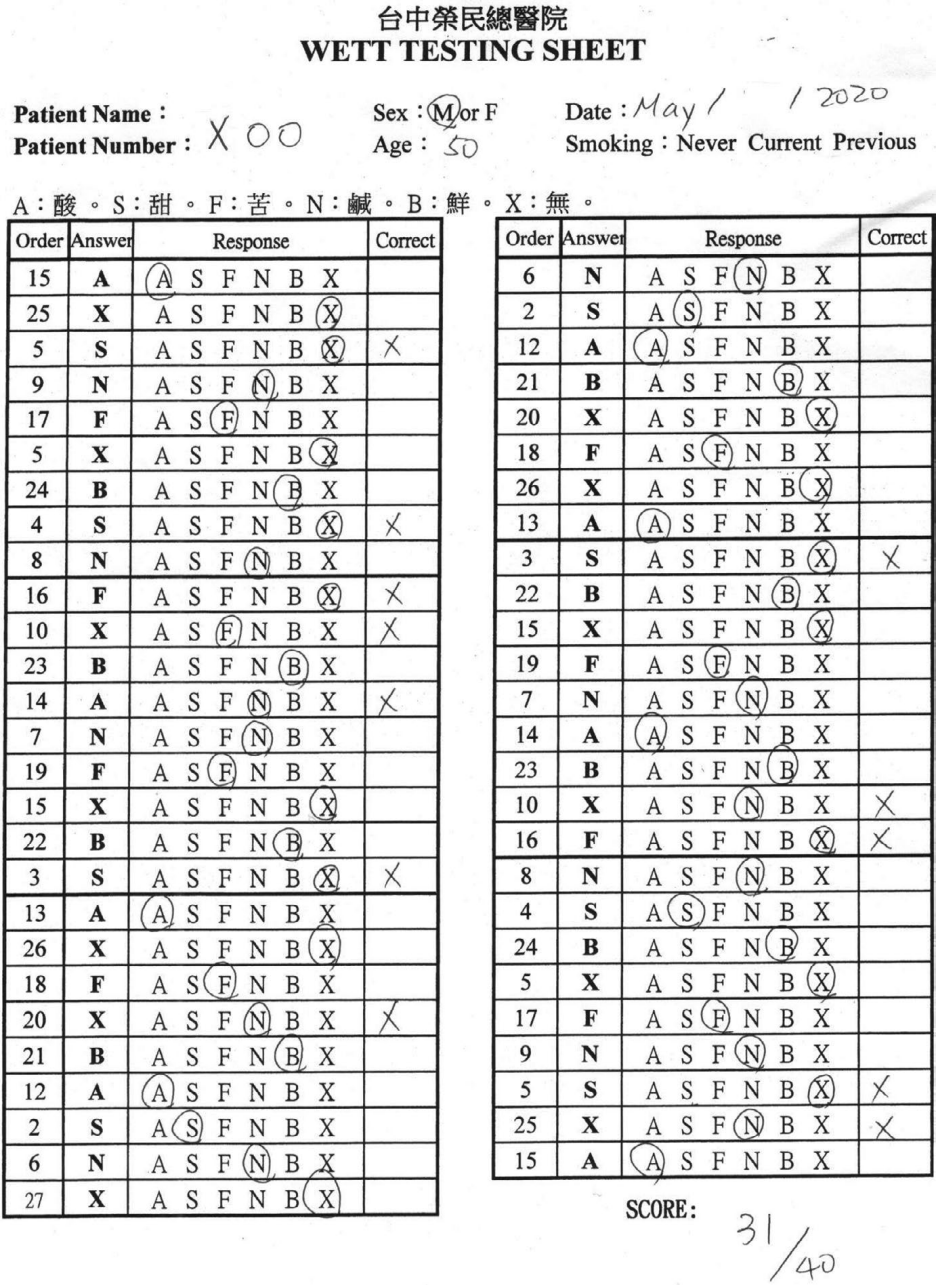
The score of the Waterless Empirical Taste Test was 31.

He later visited the Otolaryngology clinic one month after discharge. He considered that his gustatory function has returned to normal, and his olfactory function was normal. A nasal endoscopy showed that the nasal cavity was free of disease. He received a UPSIT‐TC to evaluate his olfactory function, and his score was 30 (Figure 8). He also received a WETT to evaluate his gustatory function, and the score resulted in 31 (Figure 9).

## 3 DISCUSSION

An upper respiratory tract infection is one of the most common etiologies of olfactory dysfunction.^11^ The pathophysiology of postinfectious olfactory dysfunction is unclear. Viruses may damage the olfactory neuroepithelium and receptor cells, or the olfactory central pathways such as the olfactory bulb.^12^ It is unknown which viruses are most often associated with postinfectious olfactory dysfunction, although parainfluenza virus type 3 is most likely to be a causative agent,^13^, ^14^, ^15^ while coronaviruses have not been considered as one of the possible causative viruses.^16^ Spontaneous recovery of olfactory function has been observed in one‐third of patients within two to three years of infection, but the loss of smell can remain stable in the remaining patients.^17^, ^18^ On the other hand, an upper respiratory tract infection is not one of the most common etiologies of gustatory dysfunction.^19^

It has been reported that the frequency of olfactory dysfunction in COVID‐19 patients ranges from 22% to 68%, with the frequency of gustatory dysfunction ranging from 20% to 33%.^2^ The pathophysiology of olfactory and gustatory dysfunctions in COVID‐19 patients remains unclear, but the damage to the olfactory neuroepithelium, or olfactory central pathways may be possible reasons surrounding olfactory dysfunction.^11^, ^18^ The possible reasons regarding gustatory dysfunction include changed expression of angiotensin‐converting enzyme‐2 in taste organs after salivary gland infection by the severe acute respiratory syndrome coronavirus 2.^20^, ^21^ However, several other factors such as gene regulatory networks, tissue pH, hormones, concentration of co‐factors and metabolic events may change the expression of angiotensin‐converting enzyme‐2 (SARS‐CoV‐2).^22^ It has been emphasized that the olfactory and gustatory dysfunctions in most COVID‐19 patients have been subjective in nature, and it remains unknown if patients have actual disturbances in their sense of smell or taste.^21^

Objective assessment of both olfactory and gustatory dysfunctions in COVID‐19 patients has been reported by Italian scholars.^23^ They found that patients had under‐reported the frequency of olfactory and gustatory dysfunctions.^24^ In turn, the scholars attempted to develop self‐administered olfactory and gustatory tests to replace the ordinary operator‐administered tests in order to more conveniently test COVID‐19 patients who had either been hospitalized or placed in home quarantine.^25^

The UPSIT‐TC and WETT used at our hospital are validated and commercially available self‐administered tests. Therefore, they are convenient as a means to remotely evaluate olfactory and gustatory functions in COVID‐19 patients. Our cases have shown that mild impairment of olfactory function may remain in recovered COVID‐19 patients who felt that they had achieved complete return of their olfactory function. This result was also found in Italian asymptomatic patients who presented an olfactory threshold at the lower limits of the norm.^25^ However, our patients displayed complete recovery of their taste functions. This may possibly be due to the rapid turnover of the taste receptor cells.^25^

The limitations of our case series are these patients did not receive objective smell and taste tests when they complained of olfactory and/or gustatory dysfunctions. Although 13.1% of COVID‐19 patients diagnosed at Taiwan have complained of olfactory or gustatory dysfunctions, no results of objective smell and taste tests have been reported.^26^ This emphasizes the importance of performing objective smell and taste tests in COVID‐19 patients because accurate evaluation of olfactory and gustatory functions may help to understand the pathophysiology of COVID‐19 and diagnose as well as follow‐up these patients. The second limitation is the small number of patients in our case series because the physicians did not refer COVID‐19 patients to receive smell and taste tests.

## CONFLICT OF INTEREST

None declared.

## AUTHOR CONTRIBUTIONS

PYL: treated the patients and referred to administer the test. RSJ: tested the patients and wrote this article.
